# Hospital Needs

**Published:** 1912-10-19

**Authors:** 


### Hospital Needs.

### DAIRY PRODUCE.

We recommend to the notice of all hospital managers that Messrs. Aplin and Barrett again have won the gold medal at the London Dairy Show for the finest collection of British dairy produce. To show how well the quality of the produce is maintained, it should be added that this is the tenth time that Messrs. Aplin and Barrett have won this prize since 1902.

### SANITARY TOWELS, OLD AND NEW.

The many forms of sanitary towels now upon the market show that it is not as easy as is commonly supposed to supply this article with the necessary combination of absorbent, soft, and antiseptic qualities. Mrs. Evaline's Health Towelettes are sold in six sizes, at prices varying from 1s. to 2s. 6d. per packet of one dozen. An interesting novelty attaches to sizes E4 and E6, which are provided with a hank of specially absorbent cotton, which is fixed evenly on the inner surface of the paid, and is designed to secure additionally absorbent qualities. It is claimed for these that the necessary changes are rendered less frequent—a matter of importance to nurses and patients when travelling, and one which they may be recommended to try for themselves. Mrs. Evaline's Health Towelettes can be obtained from all leading drapers and chemists, or wholesale from "Mrs. Evaline," John Street, Westgate, Bradford, Yorkshire.

